# High-concentration Ge doping of (001) β-Ga_2_O_3_ by halide vapor phase epitaxy beyond the practical Si doping limit

**DOI:** 10.1080/14686996.2026.2756746

**Published:** 2026-11-05

**Authors:** Yuichi Oshima, Takayoshi Oshima, Chia-Hung Lin, Kohei Sasaki

**Affiliations:** a Research Center for Electronic and Optical Materials, National Institute for Materials Science, Tsukuba, Japan; b Novel Crystal Technology, Inc., Sayama, Japan

**Keywords:** β-Ga_2_O_3_, HVPE, doping, resistivity

## Abstract

High-concentration *n*-type doping of (001) β-Ga_2_O_3_ grown by halide vapor phase epitaxy (HVPE) was investigated using Si and Ge dopants. While Si doping caused severe surface degradation when [Si] exceeded approximately 6 × 10^18^ cm^−3^, Ge doping produced homoepitaxial layers without surface degradation up to [Ge] = 1.9 × 10^19^ cm^−3^. X-ray diffraction measurements revealed that the degraded Si-doped regions contained in-plane rotated twin domains and exhibited broadened X-ray rocking curves, whereas the heavily Ge-doped layers maintained single crystallinity with crystalline quality comparable to that of the substrate. Hall-effect measurements showed that the carrier concentration (*n*) closely followed [Ge], indicating nearly complete electrical activation of the incorporated Ge donors. The electron mobility and bulk resistivity followed the previously reported carrier-concentration dependence for HVPE-grown Si-doped (001) β-Ga_2_O_3_. A minimum bulk resistivity of 11.6 mΩ cm was obtained. Furthermore, Ti/Pt/Au contacts fabricated on the Ge-doped layers exhibited a specific contact resistivity as low as 9.6 × 10^−6^ Ω cm^2^ for *n* = 1.3 × 10^19^ cm^−3^, comparable to values reported for more heavily Si-implanted β-Ga_2_O_3_. These results demonstrate that Ge enables heavily doped *n*-type (001) β-Ga_2_O_3_ layers to be grown by HVPE beyond the practical Si doping limit while preserving excellent crystalline quality. This capability enables monolithic growth of a thick drift layer together with heavily doped contact layers in a single HVPE growth run, providing a promising route toward lower-loss, lower-cost, and higher-performance β-Ga_2_O_3_ vertical power devices.

## IMPACT STATEMENT

Ge enables high-concentration n-type doping in HVPE-grown (001) β-Ga_2_O_3_ while preserving crystal quality, providing a practical route to low-resistance contact layers for high-performance, low-cost power devices.

## GRAPHICAL ABSTRACT

**Figure:**
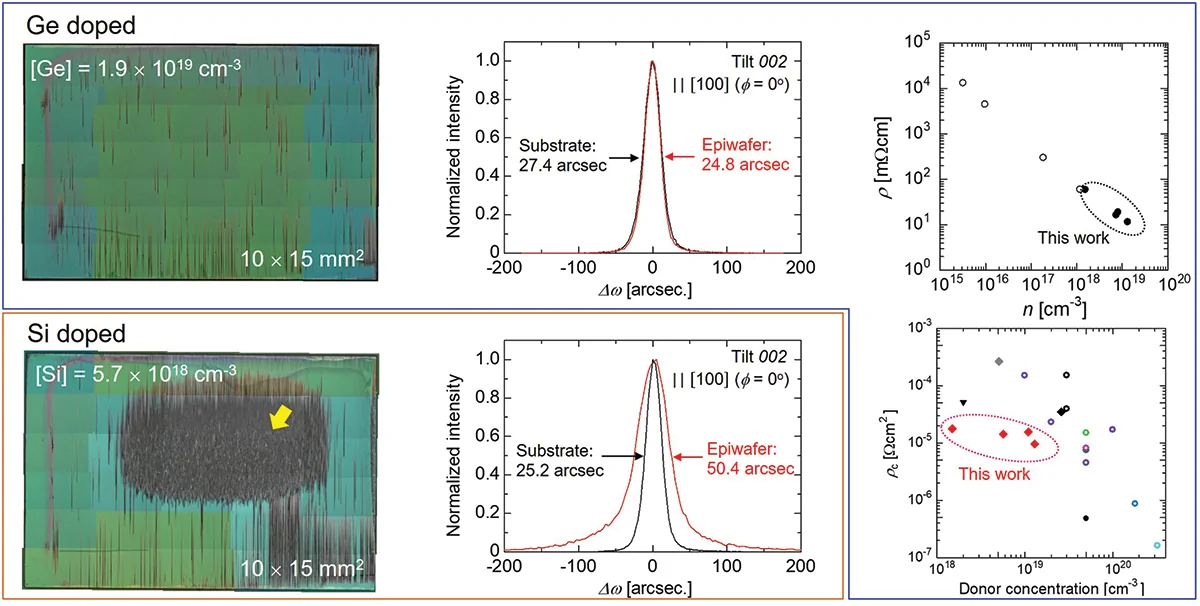

## Introduction

Monoclinic β-Ga_2_O_3_ is an ultra-wide-bandgap semiconductor with a bandgap of 4.5–4.9 eV [1,2]. Owing to its large bandgap, β-Ga_2_O_3_ is expected to possess an exceptionally high critical electric field, making it one of the most promising materials for next-generation high-power electronic devices [3]. Unlike most other wide-bandgap semiconductors, such as GaN and SiC, single-crystal β-Ga_2_O_3_ substrates can be produced by melt-growth techniques, including edge-defined film-fed growth (EFG) [4,5], the vertical Bridgman method [6–8], the Czochralski method [9,10], and the floating-zone method [11]. Consequently, high-quality homoepitaxial layers can be grown on native substrates using various epitaxial techniques, such as halide vapor phase epitaxy (HVPE) [12–16], metalorganic vapor phase epitaxy (MOVPE) [17–19], molecular beam epitaxy (MBE) [20–22], and mist chemical vapor deposition [23,24]. Owing to these unique advantages, β-Ga_2_O_3_ has emerged as an attractive candidate for next-generation power electronics, and promising device prototypes, including Schottky barrier diodes (SBDs) [25–28] and metal–oxide–semiconductor field-effect transistors (MOSFETs) [29–32], have already been demonstrated.

*n*-type doping control is one of the key technologies for realizing high-performance β-Ga_2_O_3_-based devices. Shallow donors such as Si, Ge, and Sn are commonly employed for *n*-type doping [33,34]. Depending on the device application, carrier concentrations over a wide range are required. For example, drift layers for kV-class power devices require lightly doped epitaxial layers with carrier concentrations (*n*) around 1 × 10^16^ cm^−3^ or lower [35]. In contrast, heavily doped *n*-type layers are indispensable for reducing both substrate resistance and metal/semiconductor contact resistance.

For vertical β-Ga_2_O_3_ power devices, electric current flows through the substrate. Therefore, the electrical resistance of the substrate should be as low as possible. In practical device fabrication, the substrate is typically thinned to less than approximately 100 μm by backside lapping to reduce the electrical and thermal resistance [35]. In many cases, commercial β-Ga_2_O_3_ substrates are grown by the EFG method using Sn as the donor impurity. Although *n* as high as approximately 1.1 × 10^19^ cm^−3^ has been reported [5], maintaining high crystalline quality practically limits the achievable *n* to the mid-10^18^ cm^−3^ range [35]. Consequently, further reduction of the bulk resistivity (*ρ*) is difficult. This limitation also restricts the minimum achievable specific contact resistivity (*ρ*_c_) of backside electrodes in the absence of any backside treatment.

Low-resistance ohmic contacts are equally important for minimizing the on-state resistance of β-Ga_2_O_3_ power devices. Typical examples include the source/drain electrodes of MOSFETs and the cathode electrodes of SBDs. To prevent the contact resistance from dominating the total device resistance, *ρ*_c_ should be on the order of 10^−5^ Ω cm^2^ or lower, while values in the 10^−6^ Ω cm^2^ range are highly desirable [36]. Heavy *n*-type doping effectively promotes electron tunneling at the metal/semiconductor interface and is therefore an effective approach to reducing *ρ*_c_.

To date, ion implantation has been the most widely used technique for forming heavily doped contact layers. Ti/Au contacts on Si-implanted (010) β-Ga_2_O_3_ have exhibited *ρ*_c_ values in the 10^−5^ − 10^−6^ Ω cm^2^ range [29,37–40]. For example, Sasaki *et al*. reported a very low value of *ρ*_c_ = 4.6 × 10^−6^ Ω cm^2^ for Ti/Au contacts on (010) β-Ga_2_O_3_ implanted with Si at 5 × 10^19^ cm^−3^ [37]. Tetzner *et al*. reported an exceptionally low value of *ρ*_c_ = 4.8 × 10^−7^ Ω cm^2^ for Ti/Au contacts on (100) β-Ga_2_O_3_ implanted with Ge at 5 × 10^19^ cm^−3^ [41].

Regrowth of heavily doped β-Ga_2_O_3_ has also been investigated [42–45]. For example, Alema *et al*. regrew heavily doped *n*-type (010) β-Ga_2_O_3_ by MOVPE with *n* = 3.23 × 10^20^ cm^−3^ and achieved an extremely low *ρ*_c_ value of 1.62 × 10^−7^ Ω cm^2^ using Ti/Au/Ni contacts [44].

Although most reported vertical β-Ga_2_O_3_ power devices employ the (001) orientation [35], studies on ohmic contacts to the (001) surface remain limited compared with those on the (010) surface. One reason might be that homoepitaxial layers grown on (001) substrates often exhibit rough surface morphologies, requiring chemical mechanical polishing (CMP) before electrical characterization, which complicates the fabrication process [35]. Favela *et al*. reported *ρ*_c_ = 3.48 × 10^−5^ Ω cm^2^ for Ti/Au contacts formed on a Si-doped β-Ga_2_O_3_ layer (*n* = 2.6 × 10^19^ cm^−3^) deposited on a (001) substrate by pulsed laser deposition [45]. In contrast, Ti/Au contacts formed directly on a commercially available (001) substrate with *n* = 5.1 × 10^18^ cm^−3^ exhibited a considerably higher value of 2.64 × 10^−4^ Ω cm^2^ [45].

Although ion implantation and regrowth of a heavily *n*-type contact layer have successfully reduced contact resistance, both approaches require additional processing after growth of the device epitaxial layers, increasing fabrication cost. In addition, ion implantation requires post-implantation annealing to recover implantation-induced crystal damage.

HVPE has been widely employed for growing the thick drift layers required for vertical β-Ga_2_O_3_ power devices because of its high growth rate [35]. If heavily doped *n*-type layers could also be grown by HVPE, they could be formed in the same growth run as the device epitaxial layers, eliminating additional processing steps. Moreover, a thick heavily doped *n*-type layer could first be grown directly on the substrate before the drift layer. During the device fabrication process, the original substrate could be completely removed by backside lapping, leaving the heavily doped HVPE-grown layer as the device backside. Such a structure would simultaneously reduce both the bulk resistance and the backside contact resistance. Despite these advantages, *n*-type doping of (001) β-Ga_2_O_3_ by HVPE has so far been reported only up to approximately *n* ≈ [Si] = 1.2 × 10^18^ cm^−3^ [46].

In this work, we investigate high-concentration *n*-type doping of (001) β-Ga_2_O_3_ grown by HVPE using both Si and Ge dopants. We systematically compare the achievable doping range and the influence of dopant species on crystalline quality in the range of *n* = 10^18^–10^19^ cm^−3^. The electrical properties of the resulting epitaxial layers, including *n*, electron mobility (*μ*), *ρ*, and *ρ*_*c*_, are also evaluated. Our results reveal the origin of the previously reported Si doping limit in HVPE-grown (001) β-Ga_2_O_3_ and demonstrate, for the first time, successful Ge doping by HVPE. Ge is shown to enable substantially higher donor concentrations than Si while preserving excellent crystalline quality, providing a practical route for monolithic growth of heavily doped contact layers together with a thick drift layer in a single HVPE process. This capability offers a promising approach for simultaneously reducing device resistance and fabrication cost in future β-Ga_2_O_3_ vertical power devices.

## Experimental methods

β-Ga_2_O_3_ homoepitaxial layers were grown on 10 × 15 mm^2^ (001) β-Ga_2_O_3_ substrates (Novel Crystal Technology, Inc.) using a laboratory-built HVPE reactor operated at atmospheric pressure. The growth temperature was 1030°C. O_2_ (>99.99995% purity) and GaCl were supplied as precursors with partial pressures of 1.25 kPa and 125 Pa, respectively. GaCl was generated *in situ* by reacting metallic Ga (>99.99999% purity) with HCl gas (>99.999% purity) at 610°C in the upstream zone of the reactor. To improve precursor utilization by suppressing parasitic reactions, HCl gas was additionally introduced directly into the growth zone at a partial pressure of 188 Pa, as reported previously [15,16,47]. N_2_ (dew point < −110 °C) was used as the carrier gas. Under these conditions, the homoepitaxial growth rate was 24 μm h^−1^. Si doping was achieved using either SiH_2_Cl_2_ or SiCl_4_ as the Si source. For Ge doping, GeCl_4_ vapor generated by bubbling through liquid GeCl_4_ was introduced into the reactor.

The surface morphology of the homoepitaxial layers was examined by Nomarski optical microscopy using BX51 (OLYMPUS, Japan) and scanning electron microscopy (SEM) using SU8230 (Hitachi, Japan). Crystal orientation was confirmed by X-ray diffraction (XRD) 2*θ*–*ω* scans and pole figure measurements of the *400* diffraction. Crystalline quality was evaluated by X-ray rocking curve (XRC) measurements of the symmetric *002* diffraction and the skew-symmetric *400* diffraction. X’Pert PRO MRD (PANalytical, Holland) was used for the XRD measurements. [Si] and [Ge] were determined by secondary ion mass spectrometry (SIMS).

*n*, *μ*, and *ρ* were measured at room temperature by the van der Pauw Hall method using ResiTest8300 (TOYO Corporation, Japan). Ti (80 nm)/Pt (20 nm) Hall contacts were deposited by electron-beam (EB) evaporation, followed by rapid thermal annealing (RTA) at 550°C for 1 min in N_2_ gas flow. *ρ*_c_ was evaluated at room temperature using the circular transmission line method (CTLM) using a semiconductor parameter analyzer B1500A (Keysight, US). Ti (30 nm)/Pt (20 nm)/Au (70 nm) electrodes were deposited by EB evaporation, followed by RTA at 550°C for 1 min in N_2_ gas flow. The contact diameter was 200 μm, and the gap spacing ranged from 4.5 to 34.5 μm. In donor-doped β-Ga_2_O_3_ epilayers, post-growth annealing has commonly been performed to activate the dopant and reduce the resistivity [46,48]. Prior to electrical characterization, all samples were subjected to an activation anneal followed by CMP. The activation anneal was carried out immediately after epitaxial growth by maintaining the samples at the growth temperature in N_2_ gas flow for 1 hr in the HVPE reactor.

## Results and discussion

Figure 1 shows [Si] and [Ge] as a function of the dopant precursor supply ratio, *R*_*x*_, defined as *R*_*x*_ = *P*_*x*_/(*P*_*x*_ +*P*_*GaCl*_), where *P*_*x*_ (*x* = SiCl_4_, SiH_2_Cl_2_, or GeCl_4_) and *P*_GaCl_ are the partial pressures of the dopant precursor and GaCl supply, respectively. Figures 2(b–f) show Nomarski optical micrographs of approximately 6-μm-thick Si- and Ge-doped homoepitaxial layers. The labels in Figure 2(b–f) correspond to those in Figure 1. Figure 2(a) shows an unintentionally doped (UID) layer grown without introducing a dopant precursor. [Si] indicated in Figure 2(a) corresponds to the background impurity level, which is most likely attributable to the use of a quartz reactor tube. The line-shaped macroscopic defects extending along the [010] direction in Figure 2, which are also observed on the surface of the UID sample, originate from pre-existing substrate defects, foreign particles, or particles deposited during epitaxial growth, and are therefore unrelated to the *n*-type doping investigated in this work.

**Figure 1.**
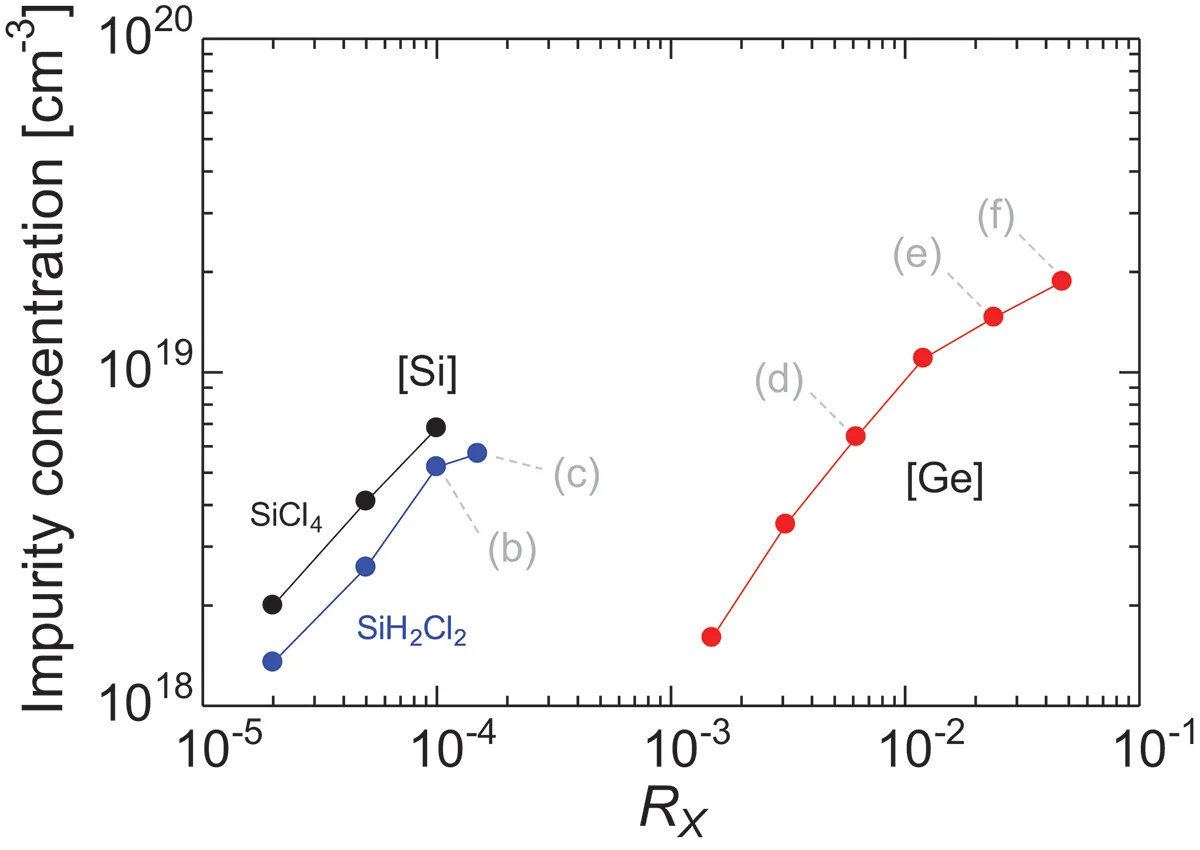
Dependence of [Si] and [Ge] on the dopant precursor supply ratio,*R_x_* = *P_x_*/(*P_x_* + *P*_GaCl_) where *P_x_*(*x* = SiCl_4_, SiH_2_Cl_2_, or GeCl_4_) and*P*_GaCl_are the partial pressures of the dopant precursor and GaCl supply, respectively. The labels (b)–(f) correspond to those used in Figures 2 and 3.

**Figure 2.**
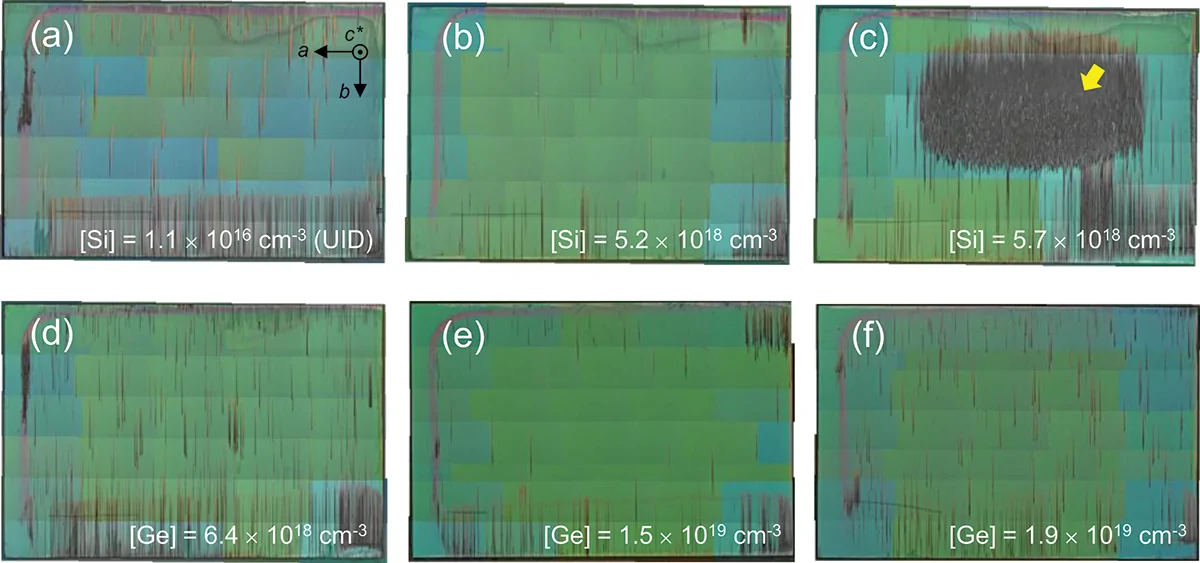
Nomarski optical micrographs of the entire surfaces (10 × 15 mm^2^) of β-Ga_2_O_3_epitaxial wafers with various [Si] and [Ge]. Si doping was performed using SiH_2_Cl_2_.

**Figure 3.**
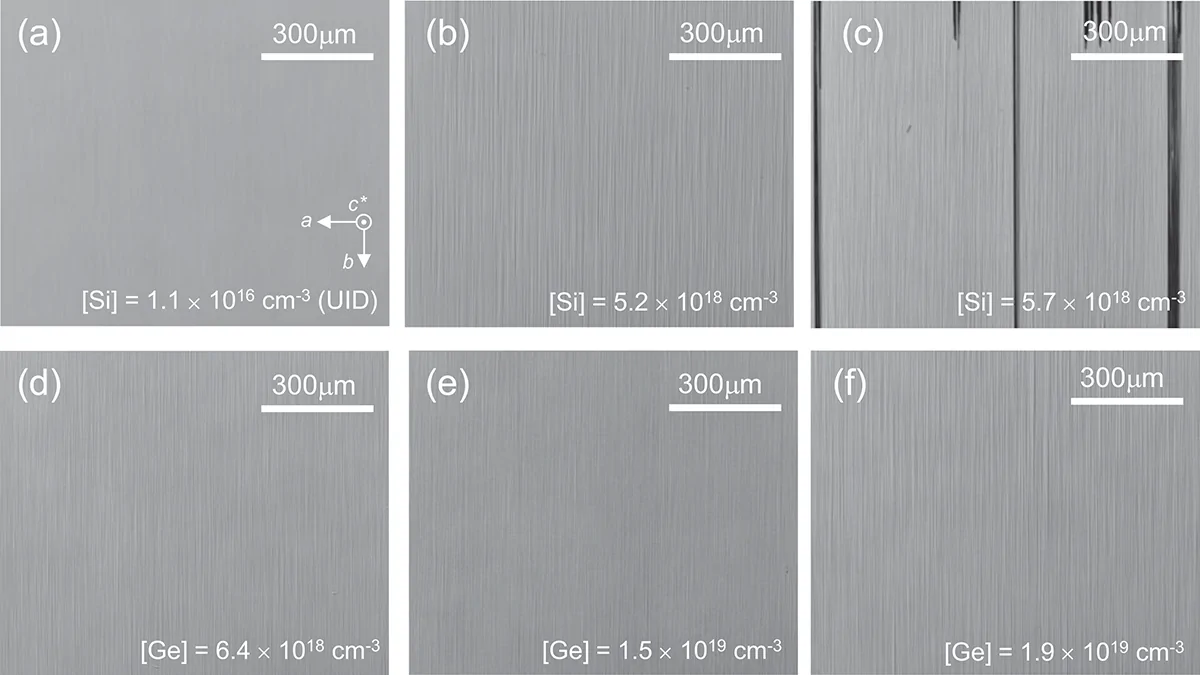
Magnified Nomarski optical micrographs of β-Ga_2_O_3_epitaxial layers with various [Si] and [Ge]. Si doping was performed using SiH_2_Cl_2_.

[Si] increased monotonically with increasing *R*_*x*_. However, pronounced surface morphological defects appeared when [Si] reached 6.8 × 10^18^ cm^−3^ for SiCl_4_ and 5.7 × 10^18^ cm^−3^ for SiH_2_Cl_2_, as indicated by the arrow in Figure 2. It should be noted that these [Si] were measured in the morphologically normal regions adjacent to the defective areas. Accordingly, the highest [Si] achieved without noticeable surface degradation were 4.1 × 10^18^ cm^−3^ for SiCl_4_ and 5.2 × 10^18^ cm^−3^ for SiH_2_Cl_2_. In contrast, [Ge] also increased monotonically with increasing *R*_*x*_ and reached 1.9 × 10^19^ cm^−3^ without any observable surface degradation.

A similar contrast between Si and Ge doping has previously been reported for HVPE-grown GaN [49]. In that study, severe surface degradation under heavy Si doping was attributed to the formation of stable silicon nitride species through reactions between SiH_2_Cl_2_ and NH_3_, which suppressed crystal growth. In contrast, the corresponding germanium nitride species were considered much less stable and therefore did not inhibit growth.

Although the growth chemistry differs substantially between GaN and β-Ga_2_O_3_, the observed contrast between Si and Ge suggests that an analogous mechanism may operate in the present system. In β-Ga_2_O_3_ HVPE, the difference in surface morphology may be associated with the thermodynamic stability of Si- and Ge-containing oxide species formed through reactions with O_2_. For example, the Gibbs free energies of formation of SiO_2_ and GeO_2_ at the growth temperature (1030 °C) are −678 and −329 kJ mol^−1^, respectively [50], indicating that GeO_2_ is substantially less stable than SiO_2_. Although the detailed mechanism remains to be clarified, this difference in oxide stability may influence the surface reaction kinetics during HVPE growth and thereby determine whether surface morphological degradation occurs.

Figure 1 also reveals a significant difference in doping efficiency among the dopant precursors. To obtain the same donor concentration, the required precursor supply ratio follows the order GeCl_4_ >> SiH_2_Cl_2_ > SiCl_4_, indicating that the doping efficiency follows the reverse order, SiCl_4_ > SiH_2_Cl_2_ >>GeCl_4_.

The origin of the lower Ge incorporation efficiency has not yet been clarified. In β-Ga_2_O_3_, both Si and Ge are considered to substitute for Ga sites [33]. Accordingly, the incorporation of these dopants is expected to depend, at least in part, on the stability of their bonding with oxygen at the growth front. Because the Ge–O bond is weaker than the Si–O bond [51], Ge may be less readily incorporated into the growing crystal and more likely to form volatile Ge-containing species, such as GeO. The subsequent volatilization of these species could reduce the Ge incorporation efficiency compared with that of Si. However, further experimental and theoretical investigations are required to clarify the incorporation mechanism.

The lower doping efficiency of SiH_2_Cl_2_ compared with SiCl_4_ is likely attributable to its higher chemical reactivity with O_2_. A comparison of the equilibrium constants for the reactions of SiH_2_Cl_2_ and SiCl_4_ with O_2_ is provided in Figure S1. Because SiH_2_Cl_2_ is more susceptible to gas-phase parasitic reactions, a larger fraction of the precursor is expected to be consumed before reaching the substrate surface, thereby reducing the effective Si incorporation efficiency.

Figure 3 shows magnified Nomarski optical micrographs of the homoepitaxial layers with various [Si] and [Ge]. For the sample with the highest [Si], a morphologically normal region in the vicinity of the degraded area was observed (Figure 3). Figure 3(a) corresponds to the unintentionally doped (UID) sample shown in Figure 2(a), while Figure 3(b–f) correspond to the samples labeled in Figures 1 and 2. Compared with the UID sample, the intentionally doped layers exhibited more pronounced stripe-like surface features extending along the [010] direction. However, no clear correlation was observed between the prominence of these stripe-like features and either the dopant concentration or the dopant species except for the sample with [Si] = 5.7 × 10^18^ cm^−3^ shown in Figure 3.

Figure 4 shows SEM images of the degraded region in the Si-doped sample with [Si] = 5.7 × 10^18^ cm^−3^ shown in Figure 2. The degraded region consisted of numerous macroscopic grooves elongated along the [010] direction. Based on our experience with (001) β-Ga_2_O_3_ homoepitaxy, such [010]-elongated macroscopic defects tend to develop from local disturbances on the growth surface, such as those caused by particle deposition. Therefore, the numerous grooves observed here may have developed from local disturbances or defects generated under heavy Si-doping conditions, although their microscopic origin remains unclear.

**Figure 4.**
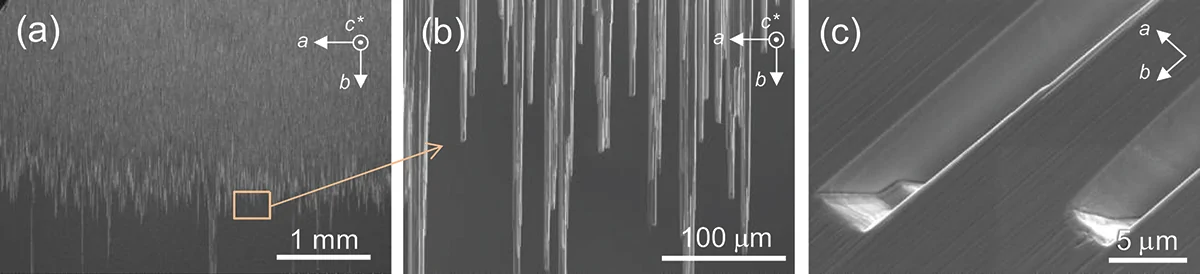
SEM images of the degraded region formed on the surface of the sample with [Si] = 5.7 × 10^18^cm^−3^. (a), (b) Plan-view images. (c) Bird’s’s-eye-view image.

Figure 5(a) shows the XRD 2*θ*–*ω* scan profile obtained from the degraded region. Only diffraction peaks corresponding to the (001) orientation were observed, indicating that the out-of-plane crystal orientation was preserved despite the severe surface degradation. In contrast, the pole figure shown in Figure 5(b) exhibited not only the strong diffraction peaks at *ϕ* = 90°, which are expected for a single-crystalline (001) β-Ga_2_O_3_ layer, but also a weak additional peak at *ϕ* = 270° (indicated by the arrow). The additional peak suggests the presence of in-plane rotated twin domains within the degraded region. The formation mechanism of these twin domains is not clear at present. One possible explanation is that the altered surface growth kinetics under heavy Si doping disturb normal epitaxial propagation and increase the probability of nucleating differently oriented domains. These locally nucleated domains may subsequently develop into the in-plane rotated twin domains observed in Figure 5(b). Further investigation is required to clarify the microscopic mechanism.

**Figure 5.**
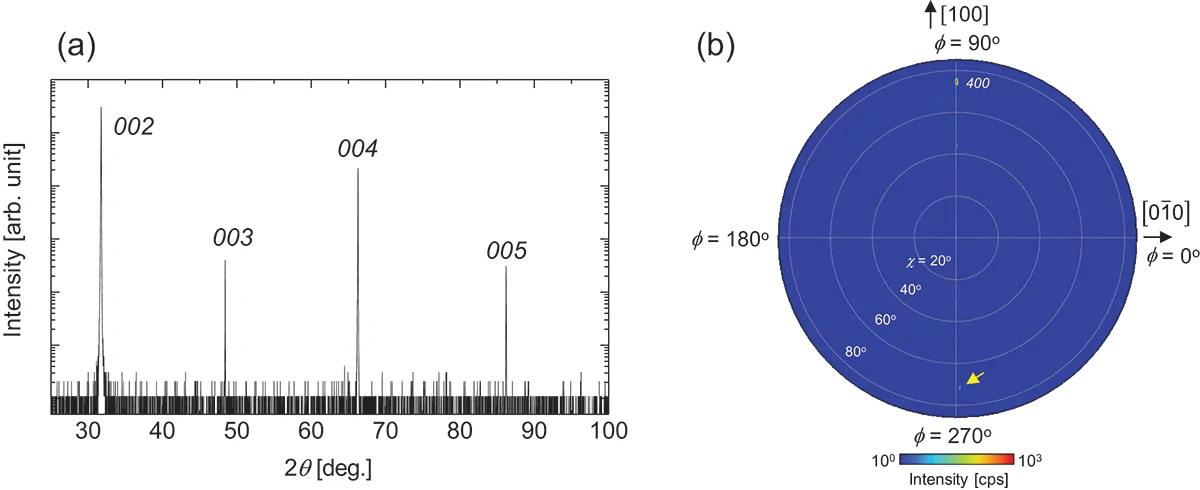
(a) XRD 2*θ–ω* scan and (b) Pole figure of the degraded region formed on the surface of the sample with [Si] = 5.7 × 10^18^cm^−3^. The arrow indicates the additional diffraction peak suggesting the presence of in-plane rotated twin domains.

Figure 6 shows the XRCs measured for the degraded region. The full-widths at half maximum (FWHMs) of both the symmetric *002* diffraction and the skew-symmetric *400* diffraction were larger than those of the substrate, indicating degradation of the crystalline quality and suggesting increased mosaicity in the heavily Si-doped layer. As discussed above for the groove formation, heavy Si doping may generate local disturbances or defects at the growth front. The increased mosaicity suggests that the influence of such disturbances is not limited to the surface morphology but extends into the epitaxial crystal, resulting in a distribution of crystallographic orientations.

**Figure 6.**
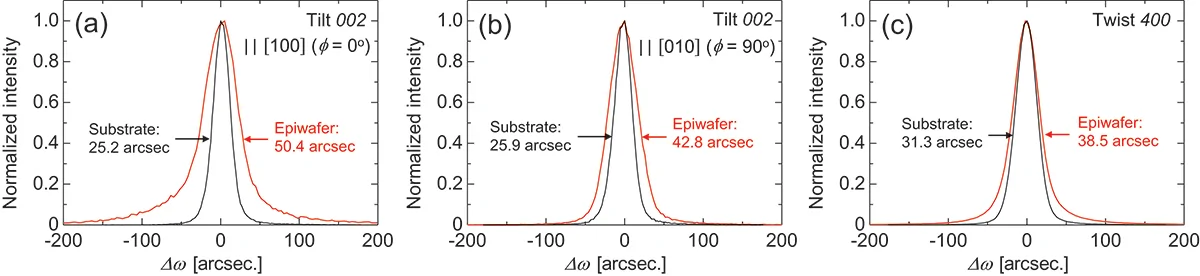
XRCs of the degraded region formed on the surface of the sample with [Si] = 5.7 × 10^18^cm^−3^.

Figure 7(a) shows the XRD 2*θ*–*ω* scan profile of the Ge-doped sample with [Ge] = 1.9 × 10^19^ cm^−3^. Only diffraction peaks corresponding to the (001) orientation were observed, indicating that the epitaxial layer maintained the intended out-of-plane crystal orientation. The corresponding pole figure is shown in Figure 7(b). In contrast to the heavily Si-doped sample, a diffraction peak was observed only at the position expected for a single-crystalline (001) β-Ga_2_O_3_ layer, with no additional peak attributable to in-plane rotated twin domains. Figure 8 shows the XRCs of the same sample. The FWHMs of both the symmetric *002* diffraction and the skew-symmetric *400* diffraction were essentially identical to those of the substrate. These results demonstrate that Ge doping enables β-Ga_2_O_3_ homoepitaxy with a [Ge] value as high as 1.9 × 10^19^ cm^−3^ while maintaining crystalline quality comparable to that of the substrate. In contrast to heavily Si-doped layers, no evidence of surface degradation, in-plane rotated twin domains, or increased mosaicity was observed.

**Figure 7.**
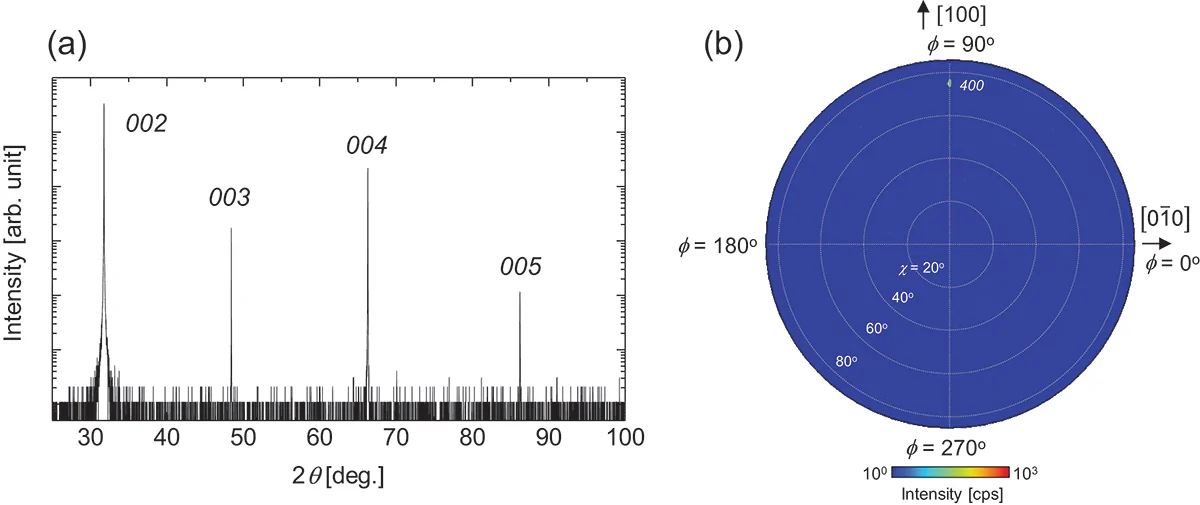
(a) XRD 2*θ–ω*scan and (b) Pole figure of the sample with [Ge] = 1.9 × 10^19^cm^−3^.

**Figure 8.**
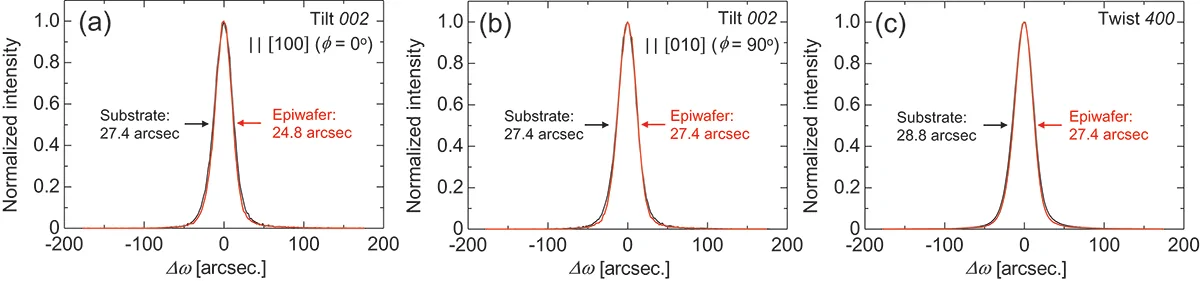
XRCs of the sample with [Ge] = 1.9 × 10^19^cm^−3^.

Figure 9(a) shows the relationship between *n* and [Ge] determined by SIMS. *n* closely followed [Ge] over the entire measured range, indicating that nearly all incorporated Ge atoms were electrically active. This observation is also consistent with the widely accepted model in which Ge acts as a shallow donor by substituting for Ga lattice sites.

**Figure 9.**
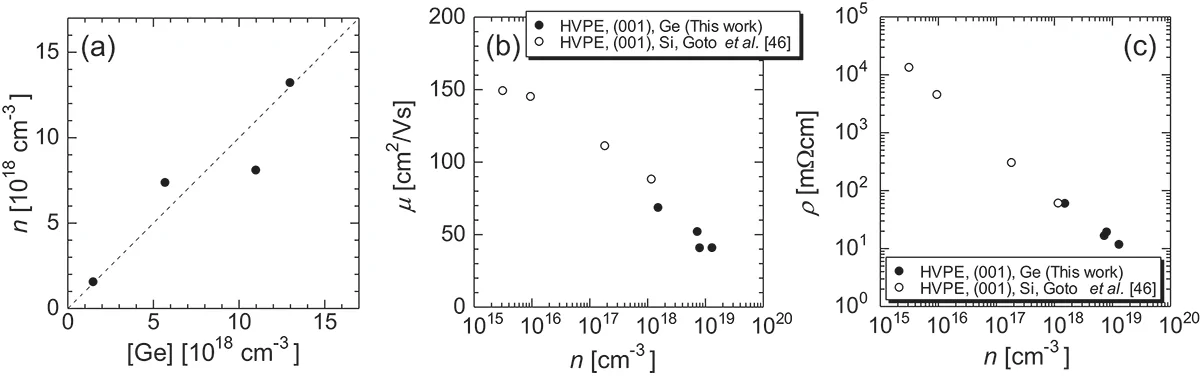
(a)*n* as a function of [Ge]. (b) *μ* and (c) *ρ* as a function of *n* for the Ge-doped samples. Previously reported data for HVPE-grown Si-doped (001) β-Ga_2_O_3_are also shown for comparison.

Figures 9(b,c) show *μ* and *ρ*, respectively, as a function of *n*. Previously reported data for HVPE-grown Si-doped (001) β-Ga_2_O_3_ are also included for comparison [46]. It should be noted that electrical characterization was not performed for the Si-doped samples prepared in the present study. *μ* and *ρ* of the Ge-doped layers followed a natural extension of the *n* dependence previously reported for HVPE-grown Si-doped (001) β-Ga_2_O_3_. This behavior, together with the XRD results presented in Figure 7 and 8, indicates that no anomalous degradation of crystalline quality occurred even at [Ge] in the 10^19^ cm^–3^ range. The lowest *ρ* value obtained in this study was 11.6 mΩ cm for the sample with [Ge] = 1.3 × 10^19^ cm^−3^. In the present study, the maximum [Ge] was limited by the maximum flow rate of the mass flow controller for GeCl_4_ supply. Therefore, a further reduction in *ρ* into the single-digit mΩ cm range may be achievable by increasing [Ge] beyond the present experimental limit.

Figure 10 shows the relationship between *ρ*_c_ and the donor (Ge) concentration of the Ge-doped layers. Previously reported data, including those for the (001) [45], (010) [29,37–40,43,44] and (100) [41,42] planes, are also plotted for comparison. Details of the CTLM measurements are provided in the Supplementary Material. The Ge-doped layers exhibited low *ρ*_c_ in the low-10^−5^ Ω cm^2^ range even at [Ge] in the 10^18^ cm^−3^ range. The lowest value obtained in this study was 9.6 × 10^−6^ Ω cm^2^ for the sample with [Ge] = 1.3 × 10^19^ cm^−3^. This value is comparable to those reported for more heavily Si-implanted (010) β-Ga_2_O_3_.

**Figure 10.**
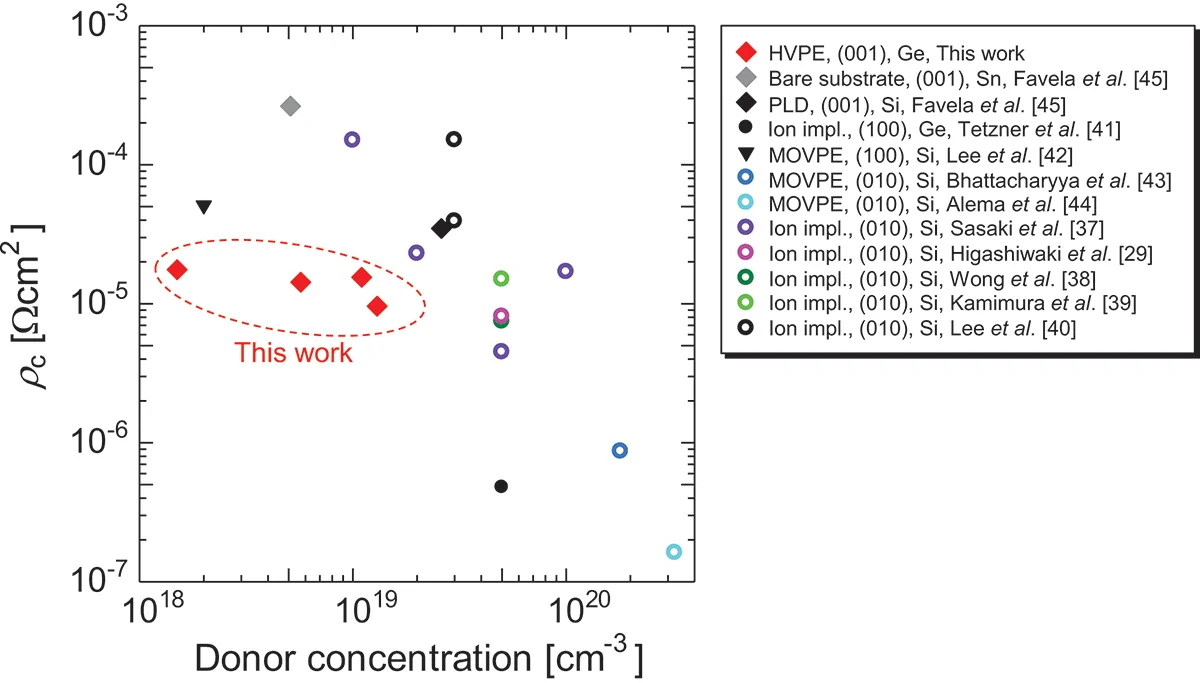
*ρ*_c_ as a function of donor (Ge) concentration for the Ge-doped samples. Previously reported data are also shown for comparison.

It should be noted, however, that the samples compared in Figure 10 differ in several important aspects, including the doping technique, crystal orientation, epitaxial growth method, and electrode structure. Therefore, direct quantitative comparison should be made with appropriate caution. Interfacial reaction layers, including Ti–TiO_*x*_ and/or defective β-Ga_2_O_3_ layers, are formed during post-deposition RTA of Ti-based contacts on β-Ga_2_O_3_ [52]. These layers are generally believed to facilitate electron transport across the metal/semiconductor interface, thereby contributing to ohmic contact formation. The formation of such interfacial layers is expected to depend on factors including crystal orientation, crystalline quality, and annealing conditions [42,45]. Since the crystalline quality of the HVPE-grown layers investigated in this work is likely different from that of previously reported MOVPE-grown layers, PLD-grown layers, or ion-implanted samples, differences in interfacial reaction kinetics may have contributed to the observed variation in *ρ*_*c*_. However, the underlying mechanism remains unclear at present and requires further investigation. Another possible contributing factor is the electrode structure. It has been reported that the optimum post-deposition annealing window for conventional Ti/Au contacts is relatively narrow and that excessive annealing can degrade the ohmic characteristics because of Au diffusion into the Ti layer [53]. In the present study, Ti/Pt/Au electrodes were employed to suppress Au diffusion [54,55], which may also have contributed to the low contact resistivity. At present, however, the relative contributions of these factors cannot be distinguished, and further studies are required to clarify the mechanism governing the contact characteristics.

## Conclusions

High-concentration *n*-type doping of (001) β-Ga_2_O_3_ by HVPE was investigated using SiCl_4_, SiH_2_Cl_2_, and GeCl_4_ as dopant precursors. For Si doping, severe surface degradation appeared when [Si] exceeded approximately 6 × 10^18^ cm^−3^, irrespective of the Si precursor employed. The degraded regions exhibited an additional pole-figure peak, suggesting the formation of in-plane rotated twin domains, together with increased XRC-FWHMs, indicating degradation of the crystalline quality. In contrast, Ge-doped layers remained free from surface degradation even at [Ge] = 1.9 × 10^19^ cm^−3^, well beyond the practical Si doping limit. X-ray diffraction measurements confirmed that these layers maintained single-crystalline growth with crystalline quality comparable to that of the substrate. Hall-effect measurements showed that *n* closely followed [Ge], indicating that nearly all incorporated Ge atoms were electrically active. *μ* and *ρ* followed the *n* dependence previously reported for HVPE-grown Si-doped (001) β-Ga_2_O_3_. Minimum values of *ρ* = 11.6 mΩ cm and *ρ*_c_ = 9.6 × 10^−6^ Ω cm^2^ were obtained for the sample with [Ge] = 1.3 × 10^19^ cm^−3^. The latter value is comparable to those reported for more heavily Si-implanted (010) β-Ga_2_O_3_. These results demonstrate that Ge enables heavily doped *n*-type (001) β-Ga_2_O_3_ layers to be grown by HVPE beyond the practical limit of Si doping while preserving excellent crystalline quality. This capability makes it possible to monolithically grow a thick drift layer together with heavily doped contact layers in a single HVPE growth run. Furthermore, such a heavily doped layer could serve as a low-resistance backside contact layer with lower *ρ* than is practically achievable in melt-grown β-Ga_2_O_3_ substrates. This approach therefore provides a promising route toward lower-loss, lower-cost, and higher-performance β-Ga_2_O_3_ vertical power devices.

## Supplementary Material

Supplemental Material
