# Supplementary material for: High-concentration Ge doping of (001) β-Ga2O3 by halide vapor phase epitaxy beyond the practical Si doping limit
Source: Sci Technol Adv Mater. 2026 Nov 5;27(1):2756746. doi: 10.1080/14686996.2026.2756746 (PMC13757889; doi:10.1080/14686996.2026.2756746)
Supplement: Supplementary file 1 [file TSTA_A_2756746_SM5419.pdf]

Fig. S1

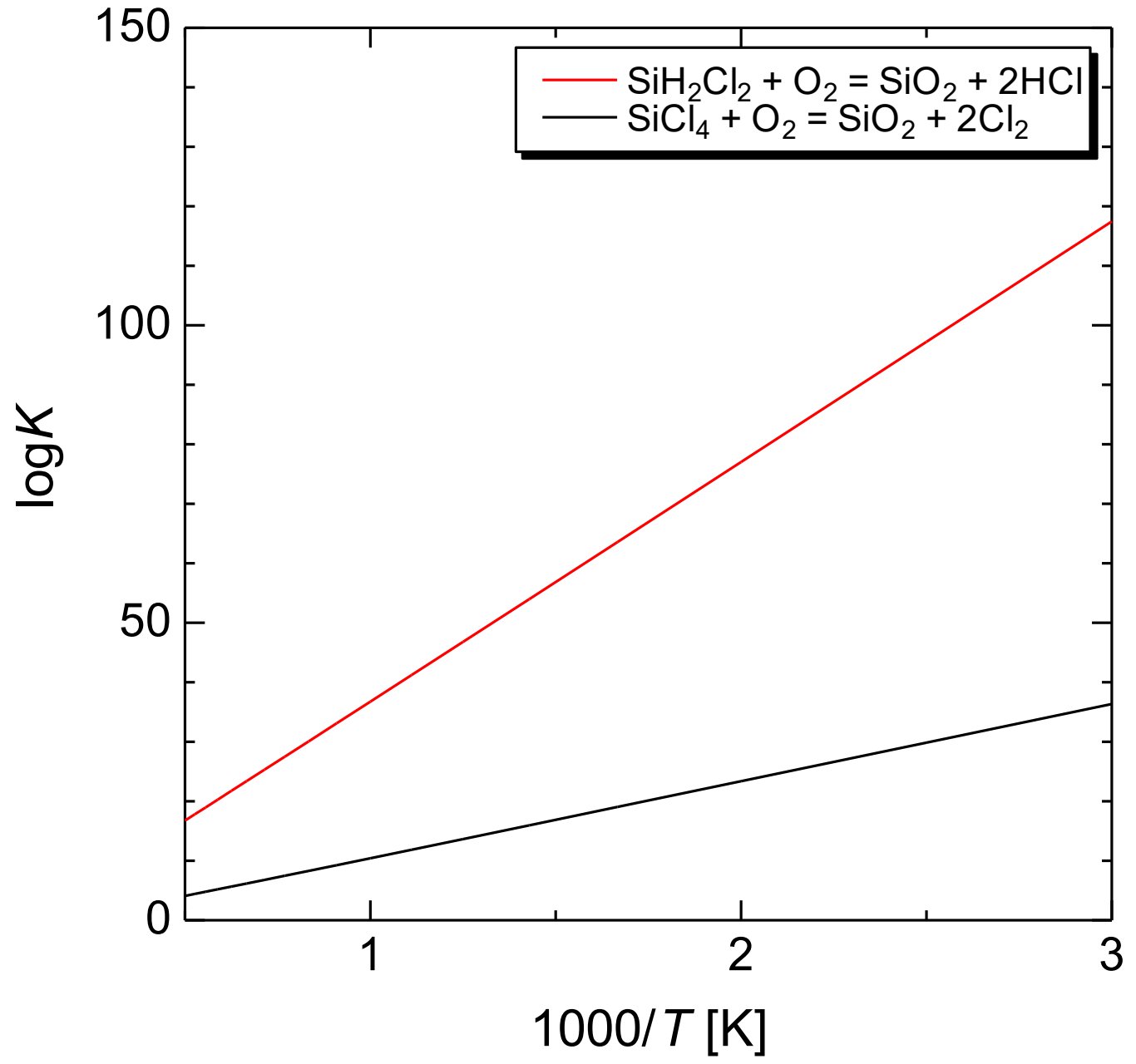

Fig. S1 Comparison of the temperature dependence of the equilibrium constants for the reactions of SiH_2Cl_2 and $SiCl_4$ with O_2. The larger equilibrium constant for SiH_2Cl_2 indicates its higher reactivity toward O_2.

Fig. S2

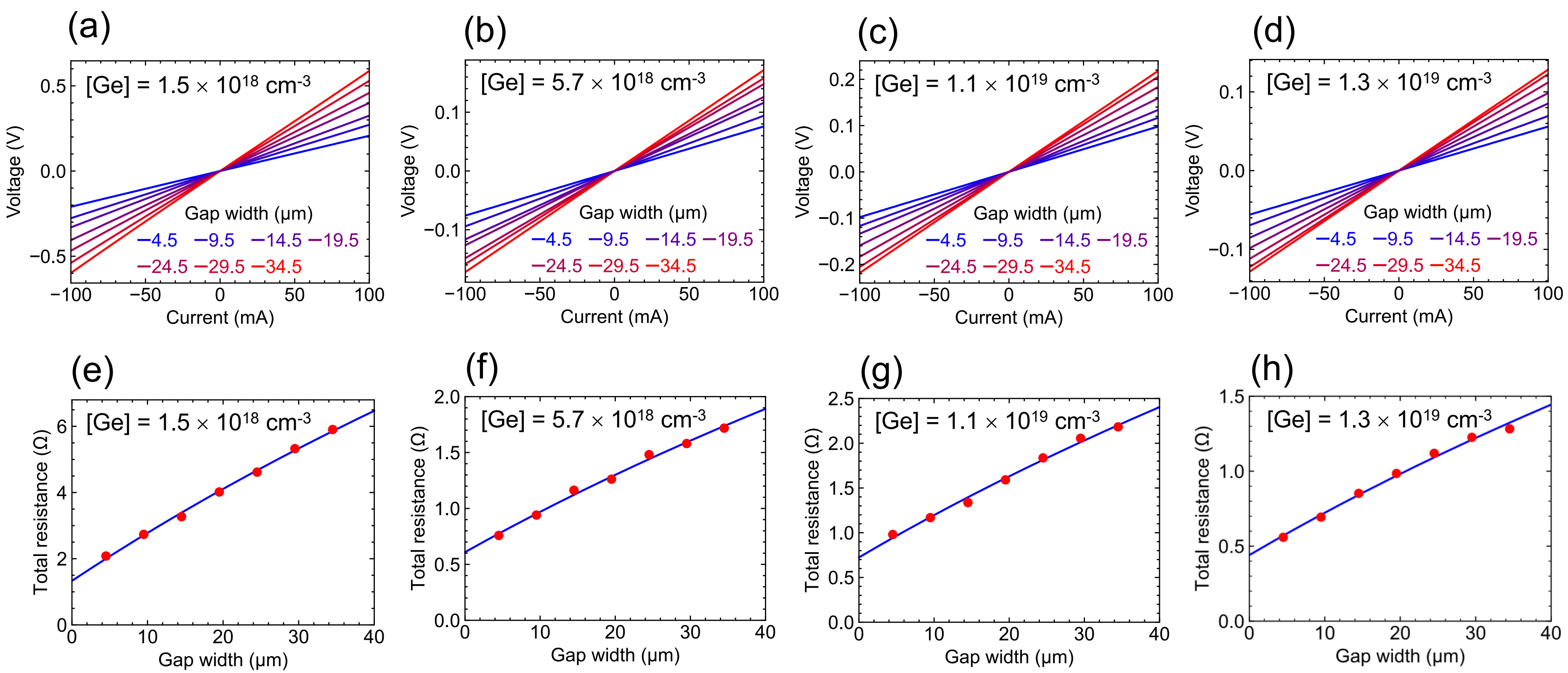

Fig. S2 CTLM measurement results for Ge-doped samples with various doping concentrations: (a)–(d) I–V characteristics and (e)–(h) total resistance as a function of gap spacing, along with fits to the theoretical model.

Table S1

Table. S1 Extracted sheet resistance, transfer length, and specific contact resistivity for Ge-doped samples with various doping concentrations, obtained from CTLM measurements.

[Ge] (cm^{-3})	Carrier conc. n (cm^{-3})	Sheet Resistance R_{sh} (Ω/sq)	Transfer Length L_T (μm)	Specific Contact Res. ρ_c (Ω·cm^2)
1.5×10^{18}	1.5×10^{18}	99.39	4.21	1.76×10^{-5}
5.7×10^{18}	7.4×10^{18}	25.46	7.49	1.43×10^{-5}
1.1×10^{19}	8.1×10^{18}	33.18	6.83	1.55×10^{-5}
1.3×10^{19}	1.3×10^{19}	19.85	6.96	9.61×10^{-6}
